# Ultra-Mutation in *IDH* Wild-Type Glioblastomas of Patients Younger than 55 Years is Associated with Defective Mismatch Repair, Microsatellite Instability, and Giant Cell Enrichment

**DOI:** 10.3390/cancers11091279

**Published:** 2019-08-30

**Authors:** Valeria Barresi, Michele Simbolo, Andrea Mafficini, Maria Liliana Piredda, Maria Caffo, Salvatore Massimiliano Cardali, Antonino Germanò, Sara Cingarlini, Claudio Ghimenton, Aldo Scarpa

**Affiliations:** 1 Department of Diagnostics and Public Health, Section of Anatomical Pathology, University and Hospital Trust of Verona, 37134 Verona, Italy; 2 ARC-Net Research Centre, University and Hospital Trust of Verona, 37134 Verona, Italy; 3 Department of Biomedical and Dental Sciences and Morphofunctional Imaging, Section of Neurosurgery, University of Messina, 98125 Messina, Italy; 4 Department of Medicine, Section of Medical Oncology, University and Hospital Trust Verona, 37134 Verona, Italy; 5 Department of Pathology and Diagnostics, Section of Pathology, Hospital Trust Verona, 37134 Verona, Italy

**Keywords:** glioblastoma, *IDH* wild-type, giant cells, mismatch repair, *POLE*, tumour mutation load

## Abstract

Background: Glioblastomas (GBMs) are classified into isocitrate dehydrogenase (*IDH)* mutants and *IDH* wild-types (*IDH*-wt). This study aimed at identifying the mutational assets of *IDH*-wt GBMs in patients aged 18–54 years for which limited data are available. Methods: Sixteen *IDH*-wt GBMs from adults < 55 years old were explored for mutations, copy number variations, tumour mutational load (TML), and mutational spectrum by a 409 genes TML panel. Results: Eight (50%) *IDH*-wt GBMs were hypermutated (TML > 9 mutations/Mb) and two (12.5%) were ultra-mutated (TML > 100 mutations/Mb). One ultra-mutated GBM had microsatellite instability (MSI), a somatic *MSH6* mutation, and a germline *POLE* mutation. The other ultra-mutated GBMs had MSI and two somatic mutations in *MSH2*. Both ultra-mutated GBMs featured at least 25% giant cells. The overall survival of eight patients with hypermutated GBMs was significantly longer than that of patients with non-hypermutated GBMs (*p* = 0.04). Conclusions: We identified a hyper-mutated subgroup among *IDH*-wt GBMs in adults < 55 years that had improved prognosis. Two cases were ultra-mutated and characterized by the presence of at least 25% giant cells, MMR mutations, and MSI. Since high TML has been associated with response to immune checkpoint inhibition in paediatric gliomas, the identification of a subtype of ultra-mutated *IDH*-wt GBM may have implications for immunotherapy.

## 1. Introduction

Glioblastoma (GBM) is the most common malignant primary tumour of the central nervous system in adults [1]. The standard of care for patients affected by GBM is maximal safe surgical resection, followed by chemotherapy with temozolomide and radiotherapy [2,3]. However, despite treatment, most GBM patients undergo recurrence and die within 15–18 months of diagnosis, while only about 5% survive more than 5 years [1].

The 2016 World Health Organization (WHO) classification subdivides GBM into isocitrate dehydrogenase *(IDH)* mutant and *IDH* wild-type (wt), based on the mutational status of *IDH1/IDH2* genes [4]. This distinction is prognostically relevant. Patients with *IDH*-mutant GBM have significantly longer overall and recurrence-free survival compared with patients with *IDH-*wt GBM [4,5,6]. Moreover, these two types of GBM have different age distributions and pathogenesis.

The *IDH*-mutant GBM is more frequent in younger patients (< 55 years, median: 45 years) and is considered to be secondary, i.e., derived from the progression of low-grade astrocytoma. In addition, it is characterized by frequent mutations in the *ATRX* and *TP53* genes [4].

The *IDH*-wt GBM mainly affects older subjects (> 55 years, median: 62 years) [7] and is considered primary, i.e., arises de novo. From a molecular standpoint, *IDH*-wt GBM is defined by the absence of *IDH* mutations and may have different genetic alterations. Among those, *EGFR* amplification is the most frequent (35–45%), followed by *PTEN* mutations/deletions and *CDKN2A* deletions [8,9,10,11].

In a recent study, about 25% of GBMs, and mainly *IDH*-wt ones, displayed microsatellite instability (MSI) [12]. This is a condition of genetic hypermutability resulting from defective DNA mismatch repair (MMR) and characterized by clustering of mutations in highly repetitive short DNA sequences, called microsatellites [13]. Microsatellite instability is a marker of defective MMR [12], which is mostly found in recurrent GBMs as a result of treatment [14].

As *IDH*-wt GBM has the lowest incidence between 18 and 55 years, most studies on *IDH-*wt GBM focused on either paediatric age or patients above 55 years [5,9,15,16,17,18,19]. Thus, the mutational spectrum of *IDH-*wt GBM has not been specifically investigated in adults between 18 and 54 years of age.

This study aimed to identify the mutational spectrum of *IDH-*wt GBMs from adult patients younger than 55 years, exploring mutations and copy number variations of 409 genes as well as tumour mutational load and mutational signatures.

## 2. Results

### 2.1. Clinico-pathological Features

Our cohort of *IDH*-wt GBMs included 16 cases out of 108 (14.8%) consecutive GBMs that had been diagnosed in patients < 55 years in a single hospital. The cohort comprised 5 female and 11 male patients (mean age 38.3 years; range: 18–49 years) (Table 1).

One patient (case 5 GL) had a breast ductal carcinoma seven years before the occurrence of GBM. Brain metastatic carcinoma was excluded by immunohistochemistry with anti-GFAP and anti-cytokeratins antibodies. No other patient had or developed other tumours during follow-up. One patient (case 4 GL) had a family history (one brother) positive for GBM. The overall survival of the patients ranged between 1 and 79 months (median: 23.5 months; mean: 28.7 months).

**Figure 1:**
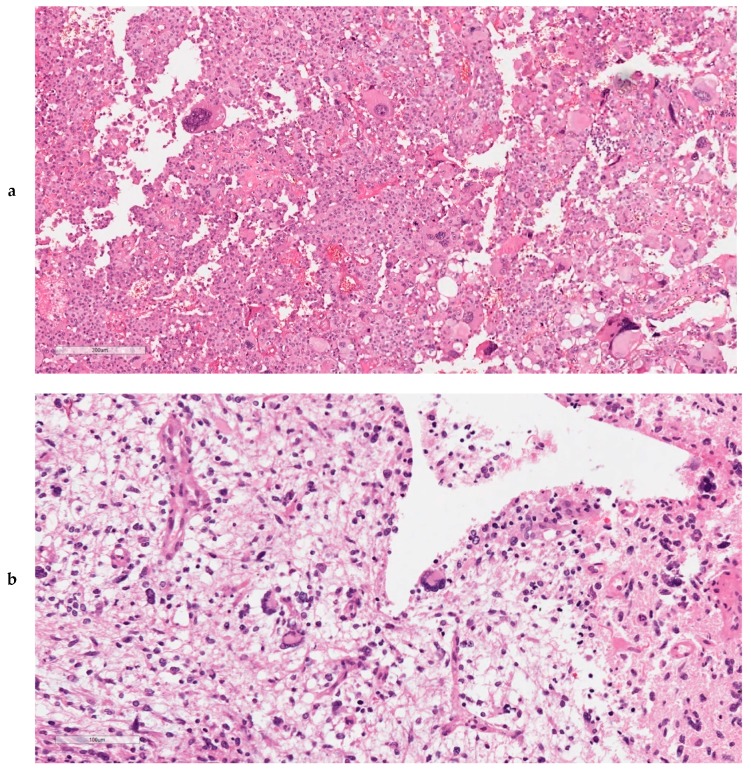
Histopathological aspects of ultra-mutated GBMs. Both cases 5 GL (**a**) and 12 GL (**b**) featured neoplastic giant cells bi- or multinucleated, which were uniformly dispersed throughout the tumour (**a**,**b**: original magnification, 200×).

At histopathology, one tumour (15 GL) was an epithelioid GBM (as defined by the presence of a dominant population of closely packed epithelioid cells) [10], one (5 GL) was a giant cell GBM (as defined by the presence of dominant bizarre multinucleated giant cells) [10], and three additional cases (1 GL, 3 GL, 12 GL) contained at least 25% multinucleated giant cells (Figure 1). In detail, in cases 1 GL, 3 GL, and 12 GL, giant cells (respectively accounting for 50%, 50%, and 25% of the tumour cells) were homogeneously distributed and intermingled with mononuclear tumour cells with milder atypia. Cases 9 GL and 16 GL also had giant cells representing less than 10% of cancer cells.

### 2.2. Mutational Status of 409 Genes

**Figure 2:**
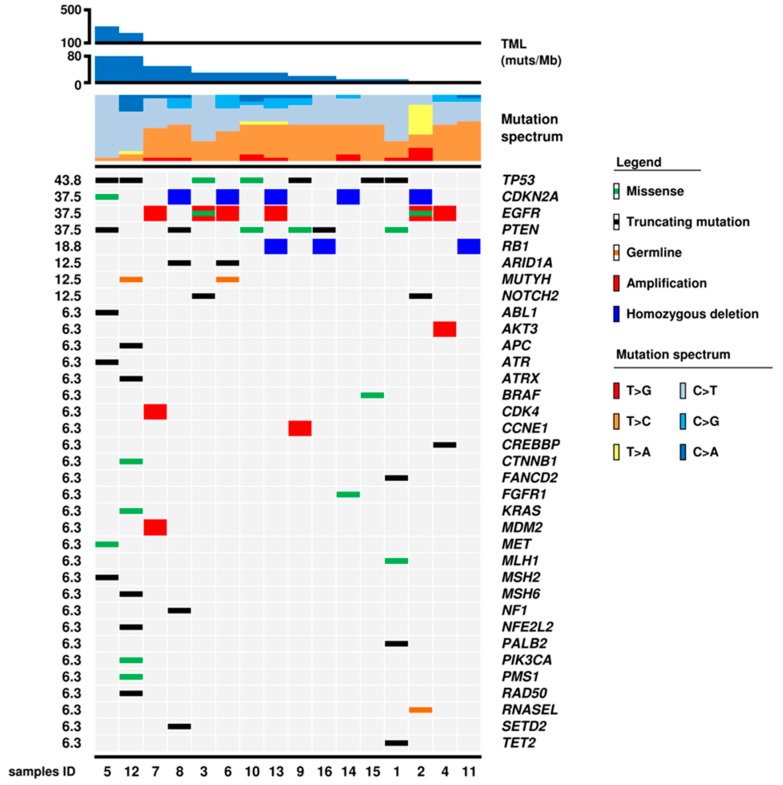
Genomic landscape of 16 *IDH*-wt glioblastomas. The matrix shows 36 genes that were altered at sequencing analysis; molecular alterations are annotated as illustrated in the panel below. Samples are ordered according to tumour mutational load (TML) from higher to lower values. The mutational spectrum is characterized by prevalent T > C and C > T transitions with low to absent contributions of T > A, T > G, C > G, and C > A transversions. The proportion of C > T over T > C increased in parallel with the increase of TML. Case 12 GL also showed a significant proportion of C > A transversions.

All 16 cases were analysed for 409 genes included in the TML assay panel (ThermoFisher, Waltham, MA, USA). Sequencing achieved an average coverage of 282× (36×–712×) in tumours and 236× (26×–651×) in normal samples (Supplementary Materials Table S1). All cases were confirmed to be *IDH1/2* wild-type. Mutations were found in at least one gene in 13 of the 16 cases, while three samples (7 GL, 11 GL, 13 GL) had no mutation in any of the 409 genes analysed (Figure 2, Supplementary Materials Table S2). Those latter cases had only mutations in untranslated regions. A total of 45 mutations in 29 genes were identified, including 19 missense, 16 nonsense, 7 frameshift, and 3 splice site alterations (Supplementary Materials Table S2).

The most frequent inactivating somatic mutations involved TP53 (7/16; 43.8%) and PTEN (6/16; 37.5%) genes, followed by MMR genes that were found altered in 3 GBMs: case 1 GL had a missense Ser193Leu mutation in *MLH1* that is classified as benign in PolyPhen database, case 5 GL had two *MSH2* mutations including a truncating Val684Ter and a missense Gly164Glu mutation, case 12 GL had a truncating Glu1322Ter mutation in *MSH6*. The *EGFR* activating Arg108Lys mutation was found in two cases (2 GL and 3 GL). A truncating Glu1365Ter mutation in the *ATRX* gene was found in one case (12 GL). A *BRAF* Val600Glu mutation was observed in the epithelioid GBM 15 GL. An *FGFR1* pathogenic missense Lys687Asp mutation was seen in case 14 GL.

Germline heterozygous mutations were found in three patients, comprising the *MUTYH* Gly396Asp (rs36053993) in patients 6 GL and 12 GL and a stop-gain (Glu265Ter) in *RNASEL* (rs74315364) in patient 2 GL (Figure 1).

### 2.3. Gene and Chromosomal Copy Number Alterations

**Figure 3:**
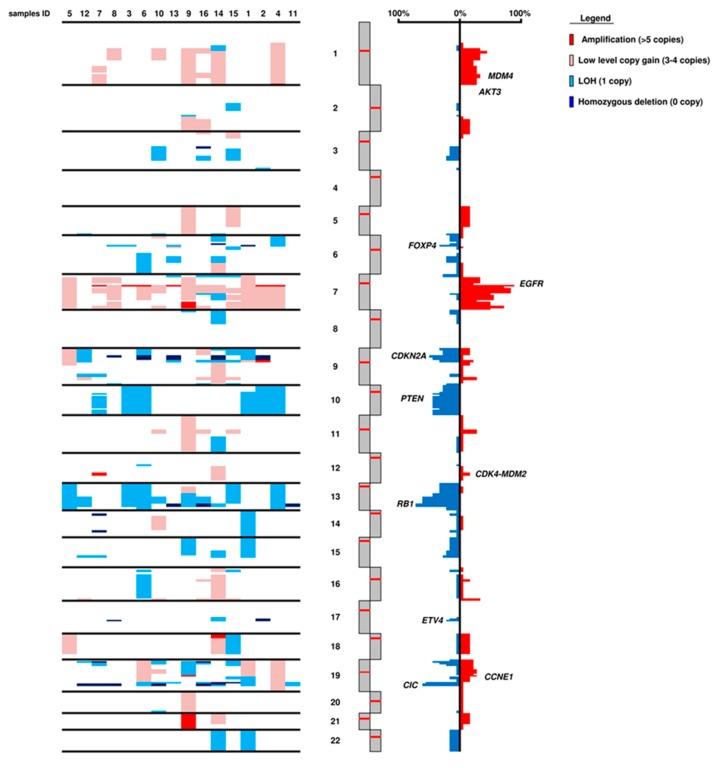
Chromosomal asset of 16 *IDH*-wt glioblastomas. The panel summarizes copy number variation (CNV) in whole chromosomes. Consensus of chromosome CNV is represented in red for copy gain events and in blue for loss events.

The CNV status was estimated for all 409 genes using sequencing data. Four genes had focal amplification: *EGFR* in 6/16 cases (37.5%), *CCNE1*, *CDK4*, and *MDM2* in 1 case each (6.3%). Two genes showed homozygous deletion: *CDKN2A* in 5/16 cases (31.3%) and *RB1* in 3/16 cases *RB1* in 3/16 cases. Based on the chromosomal position of each gene, the status of chromosome arms was inferred (Figure 3). The major alterations were gains in chromosome 7 (14/16; 87.5%) and losses in chromosomes 10 (6/16; 37.5%), 13 (12/16; 75.0%), and in 19q arm (11/16; 68.8%). Frequent homozygous deletions were detected in chromosome 19 and involved loci of *BCL3*, *CIC*, and *MARK4* genes (6/16; 37.5%).

### 2.4. Tumour Mutational Load

The number of mutations/Mb in the 16 GBMs ranged from 5.28 to 219.79 (median 8.73) (Figure 2, Table 1); 8 of the 16 cases (1 GL, 3 GL, 5 GL, 8 GL, 10 GL, 11 GL, 12 GL, 14 GL) had > 9 muts/Mb and were considered hypermutated; 2 of these (5 GL and 12 GL) had > 100 muts/Mb and were considered ultra-mutated according to Campbell et al. [20].

### 2.5. Mutational Spectrum

**Figure 4:**
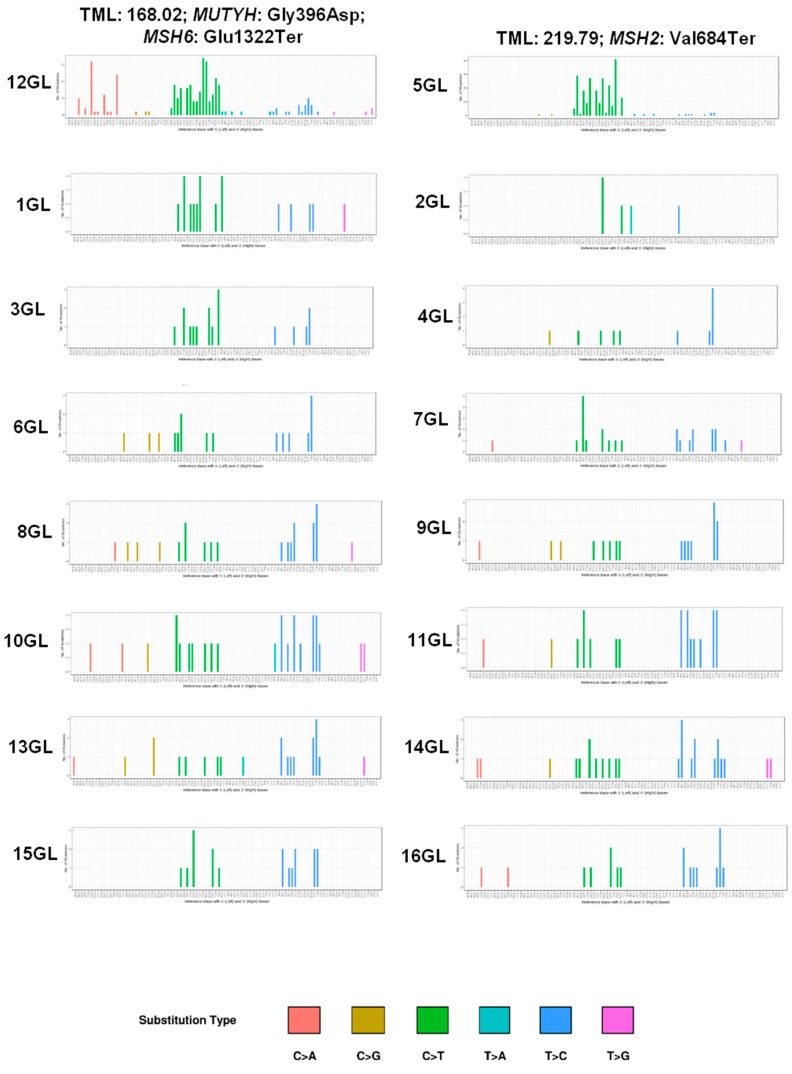
Mutational signatures of 16 *IDH*-wt glioblastomas. The signatures of somatic mutations (mutational spectrum) of individual tumours were obtained considering six major mutation classes: C > T; C > A; C > G; T > A; T > C; T > G. On top are the two ultra-mutated samples with indications of tumour mutational load (TML) and the peculiar molecular alterations. The mutational signature of case 12 GL shows a predominance of C > T over T > C transitions, and four peaks of C > A transversions in the trinucleotide contexts CCT, TCT, GCT, and ACT; this latter mutational pattern corresponds to signature SBS14 in the Catalogue Of Somatic Mutations In Cancer (COSMIC) version 3, and is associated to tumours with concurrent impairment of DNA mismatch repair and *POLE* proofreading functions (https://cancer.sanger.ac.uk/cosmic/signatures/SBS/) [25]. On the horizontal axis, the reference base with 5′ (left) and 3′ (right) bases are shown. On the vertical axis, the number of mutations is shown.

The mutational spectrum of our *IDH*-wt cohort was characterized by prevalent T > C and C > T transitions with low to absent contribution of T > A, T > G, C > G, and C > A transversions (Figure 1 and Figure 4). Notably, in addition to the predominance of C > T over T > C transitions, the ultra-mutated case 12 GL also showed a significant proportion of C > A transversions (Figure 1), which presented four peaks in correspondence of the trinucleotide contexts CCT, TCT, GCT, and ACT (Figure 2). This C > A mutational pattern accompanied to prevalent C > T transitions corresponds to signature SBS14 in the COSMIC database [21], which has been associated with the concurrent impairment of POLE and MMR functions [22].

### 2.6. POLE and POLD1 Mutations

As *POLE* and *POLD1* genes were missing from the TML assay, all samples were evaluated for mutations in these two genes using a custom next-generation sequencing panel. A germline Arg742Cys (exon 20; rs768004570) mutation was detected in the polymerase domain of *POLE* of patient 12 GL. This mutation was classified in the ClinVar database as of uncertain significance and annotated by PolyPhen software (genetics.bwh.harvard.edu/pph/) as probably damaging (Supplementary Materials Table S2).

### 2.7. Microsatellite Instability

Microsatellite instability (MSI) analysis was performed on all 16 samples comparing tumour and normal profiles of six polyA microsatellite markers. Two cases scored positive, 5 GL showed instability in 2/5 microsatellite markers (NR21, BAT26) and 12 GL in 4/5 microsatellites (NR21, NR24, BAT25, BAT26). These two cases had truncating or missense pathogenic mutations in *MSH2* and *MSH6*, respectively (Supplementary Materials Table S2). The remaining 14 cases had stable microsatellites including case 1 GL with a *MLH1* missense mutation reported as benign in the PolyPhen database (Supplementary Materials Table S2).

### 2.8. Immunohistochemical Analysis of Mismatch Repair Proteins

**Figure 5:**
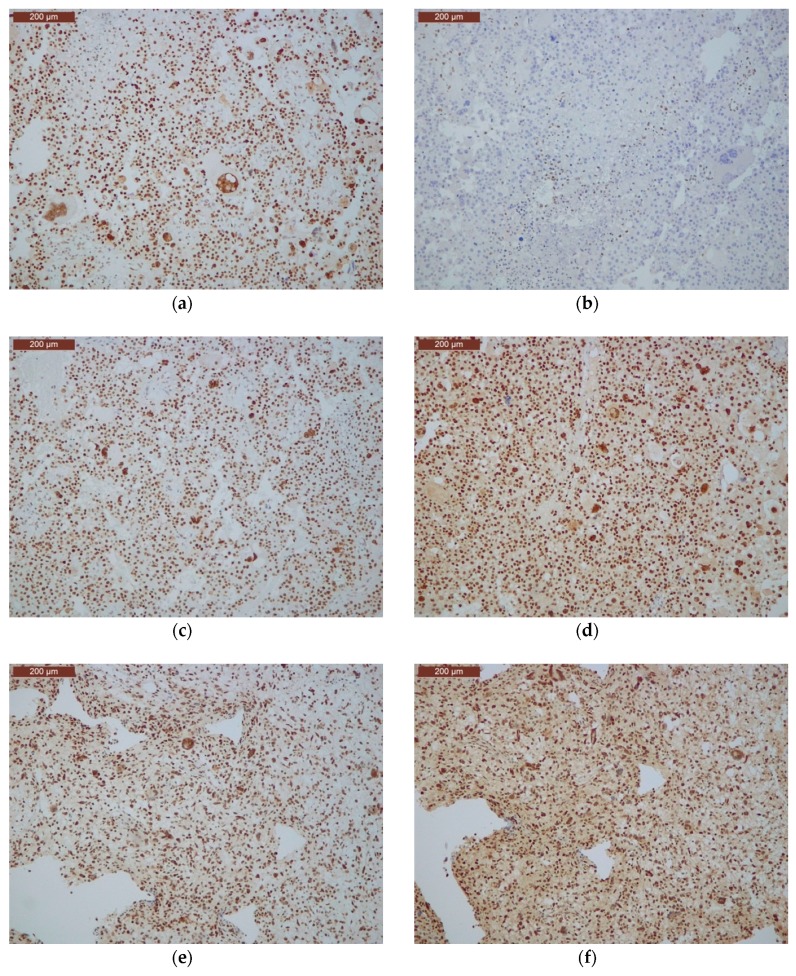

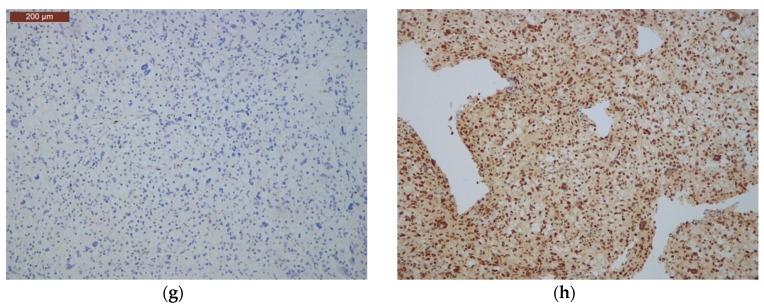
Immunohistochemical analysis of mutated mismatch repair genes in cases 5 GL (**a**–**d**) and 12 GL (**e**–**h**). Case 5 GL shows loss of MSH2 expression (**b**) and positive staining for MLH1, MSH6 and PMS2 (**a**,**c**,**d**) (original magnification, 200×). Case 12 GL shows loss of MSH6 expression (**g**) and positive staining for MLH1, MSH2, and PMS2 (original magnification, 200×) (**e**,**f**,**h**).

Three cases (1 GL, 5 GL, 12 GL) with mutations in *MMR* genes were immunostained for all four MMR proteins. Neoplastic cells of cases 5 GL and 12 GL, which respectively harboured a double mutation in *MSH2* (GLy164Glu and Val684Ter) and a mutation in *MSH6* (Glu1322Ter), showed loss of the corresponding mutated protein (Figure 5) and positive staining for the other three proteins. Case 1 GL, harbouring the missense benign mutation in *MLH1*, had positive immunostaining for all four MMR proteins.

### 2.9. Ultra-Mutated IDH-wt GBMs Featured High Content of Giant Cells, MMR Protein Loss, and MSI

Both ultra-mutated GBMs had at least 25% of giant cells at histopathology (Figure 4) and *TP53* mutations and pathogenic mutations in *MMR* genes (*MSH2* and *MSH6*); case 12 GL also had *POLE* mutation. In addition, they had microsatellite instability as detected by MSI-PCR and MMR protein loss of expression at immunohistochemistry.

### 2.10. Survival Analysis

Overall survival ranged between 3 and 79 months (median: 19 months; mean 25 months); 8 (50%) subjects died of the disease during follow-up.

**Figure 6:**
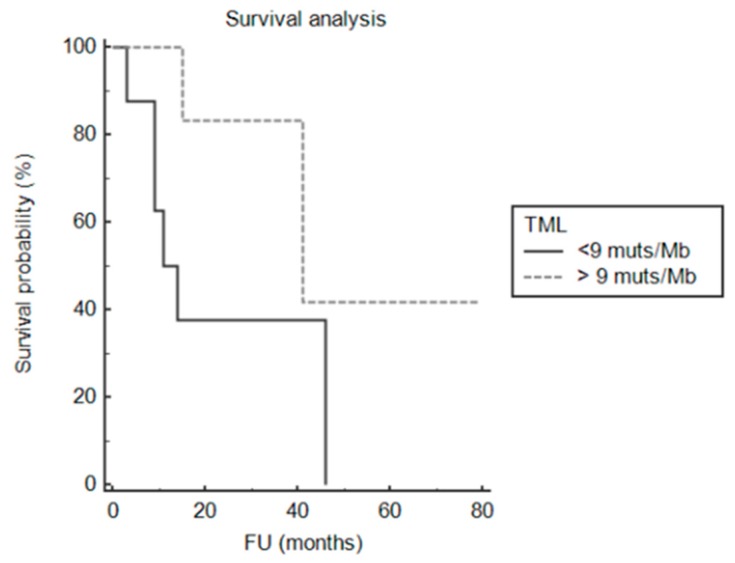
Impact of tumour mutational load on clinical outcome. Overall survival analysis showing that TML > 200 mutations/Mb was associated with better prognosis in *IDH* wild-type GBMs (*p*-value = 0.036).

At univariate analysis for tumour-specific survival, we tested: mutation in *TP53*; mutation in *PTEN*; amplification in *EGFR*; homozygous deletion in *CDKN2A*; presence of mutation in MMR genes *MSH2* and *MSH6*; presence of TML over 9 mutations/Mb. The only significant genomic alteration associated with patients’ survival was TML over 9 mutations/Mb (*p* = 0.04; Figure 6).

### 2.11. Ultra-Mutated GBMs in the TCGA PanCancer Atlas

Search for GBMs in the TGCA PanCancer Atlas through cBioPortal [23,24] identified 266 cases with available information on exome mutations and patients age. Of these, 80 patients were < 55 years (14 *IDH-*mut and 66 *IDH*-wt), and 186 ≥ 55 years (2 *IDH*-mut and 184 *IDH*-wt). In these 266 GBMs, mutations ranged from 16 to 12, 189 per exome. Eight cases could be considered hyper-mutated and two ultra-mutated according to cut offs of ≥ 9 muts/Mb for hypermutation and > 100 muts/Mb for ultra-mutation, as defined by Campbell et al. [20]. In fact, the eight hypermutated cases had ≥ 275 mutations per exome (range 275 to 1003) that correspond to > 9 muts/Mb, assuming that an exome is 1% of the genome, that is 30 × 10^6^ bp. The two ultra-mutated cases had 6910 and 12,189 mutations that correspond to > 200 muts/Mb.

Of the eight hypermutated GBMs, four were from patients < 55 years (4/80, 5%) and included three *IDH*-wt- (377, 576, and 1003 mutations) and one *IDH*-mutated case (313 mutations), while four were *IDH*-wt from patients ≥ 55 years (275, 284, 343, and 529 mutations). Among hyper-mutated GBMs, two cases had *POLE* mutations: an *IDH*-wt GBM from a 66 year old patient (TGCA-14-1795-01) which had 343 mutations and *POLE* Asp1123Asn mutation with unknown significance; a giant cell *IDH*-wt GBM from a 48 year old patient (case 19-1787-01) which had 576 mutations and *POLE* Ala399Val mutation reported of uncertain significance in the ClinVar database. None of the hypermutated cases had *MMR* mutations.

Only two cases were ultra-mutated and were *IDH*-wt from females aged 53 and 23 years, respectively: TCGA-19-5956 with 6910 mutations and TCGA-06-5416 with 12,189 mutations. Both cases harboured concomitant somatic mutations in *POLE* and *MMR* genes, namely, TCGA-19-5956 had two *POLE* (Arg1826Trp; Ala456Pro) and one *MLH1* (Arg265Cys) missense mutations; TCGA-06-5416 had a *POLE* (Val411Leu), *MSH2* (Lys871Asn), and *MSH6* (Arg482Gln) missense mutations. Notably, case TCGA-19-5956 had been classified as giant cell GBM and case TCGA-06-5416 histologically showed multinucleated giant cells.

## 3. Discussion

The results of our study on *IDH*-wt GBMs of adults < 55 years may be summarized as follows: (i) *IDH-wt* GBMs accounted for 14.8% (16/108) of GBMs of this age group; (ii) TML in these 16 cases ranged between 5.66 and 219.79 mutations per megabase and, thus, 8 (50%) GBMs were hypermutated (> 9 muts/MB) and two of those were ultra-mutated (> 100 muts/MB); (iii) 2 ultra-mutated GBMs had inactivating mutations in one MMR gene and MSI and they featured enrichment in giant cells; iv) one ultra-mutated GBM had also a germline *POLE* mutation previously undescribed as pathogenic; v) hyper-mutated GBMs had longer life expectancy.

The *IDH-wt* GBMs accounted for about 15% of GBMs < 55 years in our hospital series and were characterized by frequent *TP53* and *PTEN* mutation, *EGFR* gene amplification, *CDKN2A* homozygous deletion, gains of chromosome 7 and losses of chromosomes 10 and 13. All these figures are in line with those reported for *IDH-*wt GBMs [9,10,11,26,27,28].

As highlighted by Campbell et al., dissimilar thresholds and methodologies and the lack of an agreed definition of hypermutation are major issues in the study of TML [20]. To address these questions, they analysed TML in 78,452 adult and 2885 childhood cancers and established 9.9 and 9 muts/Mb as cut-offs for hypermutation in childhood and adult cancers, respectively, and 100 muts/Mb as the cut-off for ultra-mutation [20]. They also demonstrated excellent concordance among exome, genome, and two separate targeted panel sequencing consisting of 315 and 884 genes covering 1.1 and 3.25 Mb, respectively, for the assessment of hypermutation across different types of cancer [20]. By using those cut off values, 8 out 16 *IDH-wt* newly diagnosed GBMs in our cohort had > 9 muts/Mb and should be considered hypermutated; two of those had > 100 muts/Mb and should be classified as ultra-mutated. The frequency of hyper- and ultra-mutated tumours in our cohort (50% and 12.5%, respectively) was higher than that reported in other cohorts of GBMs [16,17] and that found in GBMs within the TGCA PanCancer Atlas [20,21] However, it is in line with the high frequency of hypermutation in brain tumours found by Campbell et al. [20] among all hypermutated and ultra-mutated neoplasms. Difference with other studies may be due to the use of different methodologies in terms of analysed genes and type of mutations considered. In addition, we may hypothesize that the high frequency of ultra-mutated GBMs could depend on the selection criteria of our cohort (i.e., *IDH*-wt status and age below 55 years). Indeed, a similar frequency (11%) of ultra-mutated cases was present among *IDH*-wt high-grade gliomas from patients < 55 years old in the cohort of Erson-Omay et al. [29]. In addition, two ultra-mutated GBMs found in the Glioblastoma Multiforme TGCA PanCancer Atlas were *IDH-*wt and were from patients < 55 years of age.

It is known that different mutagenic processes give rise to different combinations of mutation types, called signatures [30]. The mutational spectrum of our *IDH*-wt cohort was characterized by prevalent T > C and C > T transitions. Accordingly, two ultra-mutated GBMs had a signature characterized by a predominance of C > T over T > C transitions and low to absent contributions of T > A, T > G, and C > G transversions signatures [30]. Interestingly, one of the cases also featured a high rate of C > A transversions which presented four peaks in the trinucleotide contexts CCT, TCT, GCT, and ACT. This mutational signature overlaps COSMIC signature SBS14, which has been associated with tumours with concurrent impairments of POLE and MMR functions [21,22]. Fittingly, this GBM had MSI associated to a somatic *MSH6* mutation and a germline mutation in the polymerase domain of *POLE*. This latter is the Arg742Cys substitution, which is reported as of uncertain significance in the ClinVar database. It involves the polymerase domain of POLE, and not the proofreading exonuclease one, different from POLE mutations which are commonly associated with hypermutation [19]. However, the association of Arg742Cys substitution with the COSMIC SBS14 mutational signature strongly suggests its pathogenicity [18]. Indeed, this signature is characteristic of tumours showing both MMR deficiency and POLE loss of function [18]. In addition, Campbell et al. [20] recently reported *POLE* driver mutations outside the exonuclease proofreading domain, suggesting that other domains might be responsible for proofreading. Interestingly, Erson-Omay et al. previously reported six ultra-mutated high-grade gliomas with a signature characterized by an increased proportion of C > A transversions associated with somatic mutations in the *POLE* exonuclease domain and, in three cases, a germline MSH6 mutation [21]. 

Hypermutation in cancer is associated with defective MMR and/or POLE functions [16,20]. In particular, isolated MMR deficiency and microsatellite instability have been mostly restricted to hyper-mutated tumours (TML between 10 and 100 muts/Mb), while ultra-mutated tumours (>100 muts/Mb) are microsatellite stable and *POLE* mutated. This study showed that hypermutation in GBM is not a unique feature of tumours with MMR genetic alterations. Indeed, fourteen hypermutated GBMs (six in our cohort and eight in the TGCA cohort) had no *MMR* mutations. In addition, it demonstrated that ultra-mutation can also be found in GBMs with MSI and isolated MMR deficiency. Indeed, one ultra-mutated GBM had *MSH2* somatic mutation in the absence of POLE impairment. Accordingly, an ultra-mutated GBM with MSH6 somatic mutation, but not POLE mutation, was previously reported by Erson-Omay et al. [21]. In addition, *MLH1* and *MSH6* somatic mutations were recently found in one primary and four recurrent *IDH*-wt GBMs [11].

Erson-Omay et al. described a predominance of large and bizarre multinucleated giant cells in ultra-mutated GBMs with *POLE* mutations and they concluded that “somatic *POLE* altered ultra-mutated GBMs represent a subset of all giant cell GBM” [20]. In line with this observation, two ultra-mutated GBMs with *POLE* mutation in the TGCA PanCancer Atlas were histologically characterized by the presence of giant cells. However, both ultra-mutated GBMs in our cohort had giant cell enrichment and none had somatic *POLE* mutation, but rather germline *POLE* mutation or isolated *MSH2* mutation. Besides, Vande Perre et al. recently reported *POLE* germline mutations in two patients with giant cell GBM [31]. Therefore, this study suggests that ultra-mutated GBMs might represent a subgroup of giant cell GBMs, which is not only characterized by *POLE* mutations. However, giant cell enrichment is not a unique feature of ultra-mutated GBMs and no ultra-mutated cases were found in a cohort of giant cell GBMs in a recent study [32].

Due to the small number of cases, we could not analyse whether there was a difference in survival length between hyper- and ultra-mutated GBMs. Although our findings should be interpreted with caution since multivariate survival analysis could not be performed due to the low number of patients, the hyper-mutated (including the ultra-mutated) GBMs in our cohort had significantly better prognosis than non-hypermutated tumours. Therefore, although *IDH*-wt GBMs are considered to have bad prognosis [4,5,6], our findings suggest that hyper-mutated GBMs may represent a less aggressive subgroup.

In addition to the prognostic significance, ultra-mutation in GBM might also have therapeutic implications. Indeed, a report suggests that ultra-mutated paediatric GBMs with germline MMR defects may have good response to immunotherapy [33]. Although limited data exist on the potential clinical benefit of immunotherapy in GBM, the evidence that 12.5% of *IDH*-wt GBM in adults < 55 years are ultra-mutated may offer alternative therapeutic strategies in this age group.

## 4. Materials and Methods

### 4.1. Cases

A total of 16 (14.8%) *IDH*-wt GBMs with available follow-up data were found among 108 GBMs that had been surgically resected from patients between 18 and 54 years at the Unit of Neurosurgery of the Azienda Ospedaliera Universitaria “G. Martino” of Messina, Italy, between 2011 and 2018. In all cases, macroscopically complete surgical removal was achieved. None of the patients received preoperative therapy. All cases were formalin-fixed and paraffin-embedded (FFPE) for routine histological evaluation. Histological diagnosis was confirmed by independent revision of two pathologists before inclusion in the study. The *IDH* mutational status was firstly assessed by immunohistochemistry against IDH1 R132H, followed by IDH1/IDH2 sequencing in immuno-negative cases. The study was approved by the Ethics Committee of the Azienda Ospedaliera Universitaria (A.O.U) Policlinico Gaetano (G.) Martino (Messina, Italy) protocol number 47/19 of 2 May 2019. Informed consent was obtained from each patient.

### 4.2. Mutational and Copy Number Variation Status of 409 Cancer Genes

For each case, DNA was obtained from FFPE tumour and from matched non-neoplastic brain (surrounding the tumour that had been removed and showing no microscopic neoplastic infiltration) using 10 consecutive 4 μm sections and the QIAamp DNA FFPE Tissue Kit (Qiagen) and qualified as reported elsewhere [34]. The Oncomine Tumour Mutational Load (TML) panel with next-generation sequencing assay (ThermoFisher) was used. The assay covers 1.65 Mb of genomic space and includes all exons of 409 cancer-related genes.

Sequencing was performed on Ion Torrent platform using 20 ng of DNA for each multiplex PCR amplification and subsequent library construction. The quality of the obtained libraries was evaluated by the Agilent 2100 Bioanalyzer on-chip electrophoresis (Agilent Technologies). Emulsion PCR to clonally amplify the libraries was performed with the Ion OneTouch™ OT2 System (ThermoFisher). Sequencing was run on the Ion Proton (ThermoFisher) loaded with Ion PI Chip v3.

Data analysis, including alignment to the hg19 human reference genome and variant calling, was done using Torrent Suite Software v.5.10 (TermoFisher). Filtered variants were annotated using a custom pipeline based on vcflib (https://github.com/ekg/vcflib), SnpSift [35], Variant Effect Predictor (VEP) [36], and NCBI RefSeq database. Additionally, alignments were visually verified with the Integrative Genomics Viewer (IGV) v. 2.3 [37] to further confirm the presence of identified mutations.

CNV was evaluated using OncoCNV v6.8 [38], comparing the BAM files obtained by sequencing of tumour samples with those obtained from blood samples. The software includes a multi-factor normalization and annotation technique enabling the detection of large copy number changes from amplicon sequencing data and permits to visualize the output per chromosome.

### 4.3. Tumour Mutational Load and Mutational Signatures

Tumour mutational load (TML) and mutational spectrum for each sample were evaluated using the Oncomine TML 5.10 plugin available on IonReporter software (ThermoFisher). Default modified parameters were used according to the manufacturer’s protocol in order to exclude false positives due to the sequencing artefacts. In detail, a threshold of at least 20 reads and an allelic frequency of 10% of variant was used to perform mutation calling. In particular, TML was expressed as the number of mutations per megabase (muts/Mb), where mutations include non-synonymous missense and nonsense single nucleotide variants (SNVs), plus insertion and deletion variants (InDels) detected per megabase (Mb) of exonic sequences.

The signatures of somatic mutations (mutational spectrum) of individual tumours were obtained considering six major mutation classes: C > T (G:C > A:T); C > A (G:C > T:A); C > G (G:C > C:G); T > A (A:T > T:A); T > C (A:T > G:C); T > G (A:T > C:G) [27,28]. Mutational Signatures in Cancer (MuSiCa) software [39] was used to obtain specific signatures for each sample. The software used .vcf files to align the sequences to the hg19 human reference genome using targeted sequencing parameters. The different types of base-pair substitutions, comprising all non-synonymous missense and nonsense SNVs were normalized per Mb of exonic sequence. The percentage of each group in each sample was computed.

### 4.4. Mutational Analysis of POLE and POLD1 Genes

A next-generation DNA sequencing custom panel was designed to investigate the mutational status of *POLE* and *POLD1* genes, which are not included in the TML panel. Sequencing was performed on Ion Torrent platform and data analysis was performed using the same pipeline used for TML panel.

### 4.5. Microsatellite Instability Analysis

MSI was tested by a fluorescent multiplex PCR exploiting the 5 mononucleotide microsatellites BAT25, BAT26, NR21, NR22, and NR24 [13]. The obtained amplicons were separated by capillary electrophoresis using the ABI Genetic Analyzer 3130XL platform (Applied Biosystems). Variations ≥ 3bp for BAT-25, NR21, and NR22 and ≥ 4bp for BAT-26 were considered as unstable.

### 4.6. Immunohistochemistry of DNA Mismatch Repair Proteins

Immunostaining was performed using the Bond Polymer Refine Detection kit (Leica Biosystems) in a BOND-MAX system (Leica Biosystems) on 4 μm thick FFPE sections using the following primary antibodies purchased from DakoCytomation: mouse monoclonal clone ES05 against MLH1 at working dilution 1:30 and clone FE11 against MSH2 at working dilution 1:30; rabbit monoclonal clone EP49 against MSH6 at working dilution 1:100, and clone EP51 against PMS2 at working dilution 1:100. Normal cells within the samples were used as positive internal controls.

### 4.7. Statistical Analysis

Overall survival was assessed by the Kaplan–Meier method, using the date of surgery as the entry data and length of survival until the patient’s death as the endpoint. Patients who died of diseases independent from GBM were censored. The Mantel–Cox log-rank test was applied to assess the strength of association between disease-specific survival and molecular alterations as a single variable. A *p*-value < 0.05 was considered as significant. All analyses were performed using MedCalc for Windows version 15.6 (MedCalc Software, Ostend, Belgium) and R v. 3.2.1

## 5. Conclusions

This study identified a molecular subgroup of ultra-mutated *IDH*-wt GBMs in adults < 55 years, which was characterized histologically by the presence of at least 25% homogenously dispersed giant cells. Ultra-mutation and giant cells were not only associated with *POLE* mutations, but also with isolated defective MMR. Although this study has the limitation of a relatively low number of cases, it opens the perspective to potentially include these patients in immunotherapy clinical trials.

## Figures and Tables

**Table 1:** Clinico-pathological features and main molecular data of 16 *IDH*-wt GBMS in adult patients younger than 55 years. Cases are ordered by increasing TML.

Case	Gender	Age	Site	OS (months)	*IDH1/2*	*EGFR*	*ATRX*	*TP53*	*PTEN*	TML	*MMR* Genes
4 GL	M	49	fronto-temporal right	DOD (11)	wt	ampl				5.28	
6 GL	M	41	fronto-temporal right	DOD (3)	wt	ampl				5.66	
2 GL	F	49	temporo-parietal left	DOD (46)	wt	ampl/mut				6.34	
15 GL	M	43	fronto-basal	DOD (14)	wt			mut		6.47	
16 GL	M	40	temporal left	DOD (9)	wt				mut	7.12	
7 GL	M	39	temporo-parieto-occipital left	DOD (14)	wt	ampl				7.33	
13 GL	F	41	temporal left	Alive (24)	wt	ampl				7.75	
9 GL	M	18	temporo-parietal left	Alive (38)	wt			mut	mut	8.22	
11 GL	F	29	cerebellar right	DOD (15)	wt					9.24	
10 GL	M	43	frontal	DOD (15)	wt			mut	mut	10.1	
8 GL	M	46	temporo-parietal left	Alive (41)	wt				mut	12.35	
1 GL	F	47	temporo-parieto-occipital right	Alive (23)	wt			mut	mut	13.07	*MLH1* mut ^*^
14 GL	M	43	frontal lobe	Alive (14)	wt			mut		14.34	
3 GL	M	24	temporal left	Alive (24)	wt	ampl/mut		mut		43.19	
12 GL	M	39	frontal	Alive (32)	wt		mut	mut		168.02	*MSH6* mut
5 GL	F	37	frontal	Alive (79)	wt			mut	mut	219.79	*MSH2* mut

OS: overall survival. DOD: died of disease. TML: tumour mutation load. MMR: mismatch repair. F: female. M: male. Ampl: amplified. Mut: mutation. * Classified as benign in the PolyPhen Database.

## Supplementary Materials

The following are available online at https://www.mdpi.com/2072-6694/11/9/1279/s1, Table S1. Coverage detail of sequencing analysis performed. Table S2. List of somatic and germline mutations identified in 13 of the 16 *IDH*-wt glioblastomas, itemized by case and alphabetical order
